# Crosstalk Between mRNA 3'-End Processing and Epigenetics

**DOI:** 10.3389/fgene.2021.637705

**Published:** 2021-02-04

**Authors:** Lindsey V. Soles, Yongsheng Shi

**Affiliations:** Department of Microbiology and Molecular Genetics, School of Medicine, University of California Irvine, Irvine, CA, United States

**Keywords:** mRNA 3' processing, epigenetics, chromatin, histone, polyadenylation

## Abstract

The majority of eukaryotic genes produce multiple mRNA isoforms by using alternative poly(A) sites in a process called alternative polyadenylation (APA). APA is a dynamic process that is highly regulated in development and in response to extrinsic or intrinsic stimuli. Mis-regulation of APA has been linked to a wide variety of diseases, including cancer, neurological and immunological disorders. Since the first example of APA was described 40 years ago, the regulatory mechanisms of APA have been actively investigated. Conventionally, research in this area has focused primarily on the roles of regulatory cis-elements and trans-acting RNA-binding proteins. Recent studies, however, have revealed important functions for epigenetic mechanisms, including DNA and histone modifications and higher-order chromatin structures, in APA regulation. Here we will discuss these recent findings and their implications for our understanding of the crosstalk between epigenetics and mRNA 3'-end processing.

## Introduction

**Figure 1:**
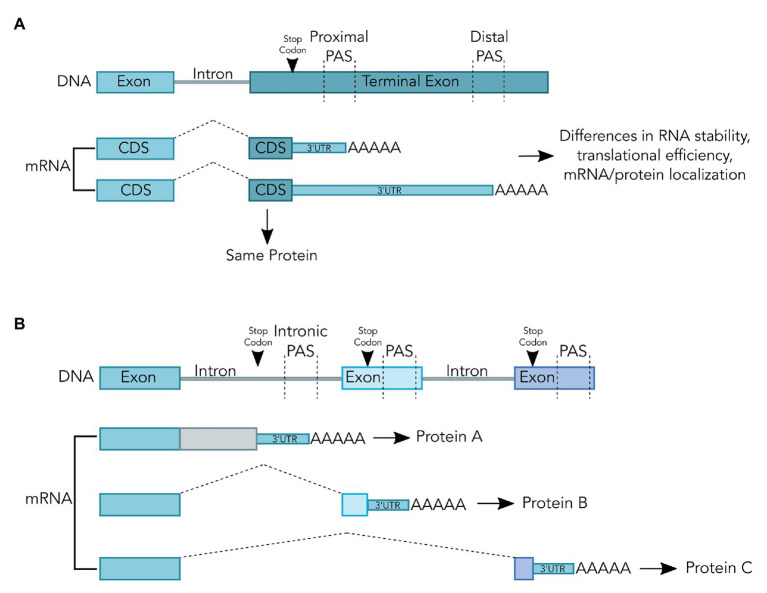
Alternative Polyadenylation (APA) can, but does not always, change the coding sequence of the resulting mRNA transcript. **(A)** APA within the terminal exon does not change the coding sequence. The poly(A) tail is shown as AAAAA and splicing is shown as dashed lines. Selection of the proximal poly(A) site (PAS) or distal PAS results in the production of mRNA isoforms with different 3'untranslated regions (3'UTRs). These mRNAs may be subject to different regulation but code for identical proteins during translation. **(B)** Alternative polyadenylation (APA) upstream of the terminal exon changes the coding sequence. The poly(A) tail is shown as AAAAA and splicing is shown as dashed lines. In the first mRNA shown, selection of the intronic PAS results in an mRNA that will produce a truncated protein if translated. This truncated protein may not be functional, which can be used to repress gene expression. In the middle mRNA isoform, selection of an alternative PAS within an alternative exon results in exclusion of the downstream exon. As a result, this mRNA isoform has a different coding sequence than the final mRNA isoform, which could produce two proteins with alternative functions.

Maturation of the 3' end for nearly all eukaryotic messenger RNAs (mRNAs) takes place in a two-step process, an endonucleolytic cleavage event followed by addition of a polyadenosine [poly(A)] tail (Colgan and Manley, 1997; Chan et al., 2011; Shi, 2012). Cleavage and polyadenylation occur at the poly(A) site, or PAS, which is recognized by the mRNA 3'-end processing machinery *via* protein-RNA interactions (Shi, 2012; Tian and Manley, 2016). A majority of eukaryotic genes use multiple alternative PAS to produce mRNA isoforms with distinct 3' ends through APA (Tian and Manley, 2016). Different APA isoforms from the same gene may differ in their the coding regions and/or the 3' untranslated regions (3' UTR; Figure 1; Tian and Manley, 2016). As such, APA can affect mRNA stability, translation efficiency, and mRNA and protein localization (Tian and Manley, 2016). APA is dynamic and highly regulated by both intrinsic and extrinsic signals. The purified human mRNA 3'-end processing complex contains both core 3' processing factors and over 50 peripheral factors that may link mRNA 3'-end processing to other cellular processes (Shi et al., 2009). However, the molecular mechanisms underlying this crosstalk remain poorly defined.

Similar to other steps of gene expression, APA is highly developmental stage- and tissue-specific. As the vast majority of the cells in multi-cellular organisms contain an identical genome, these differences are most likely caused, directly or indirectly, by epigenetic changes. Epigenetic mechanisms refer to reversible and heritable alterations that modulate gene expression without changing the DNA sequence (Cavalli and Heard, 2019). In eukaryotic cells, genomic DNA is wrapped around histone octamers to form nucleosomes, which in turn assemble into higher-order chromatin structures. Epigenetic changes could occur at multiple levels. First, DNA can be chemically modified. One of the most commonly found DNA modifications is methylation of cytosines, typically followed by a guanine nucleotide (CpG; Miranda and Jones, 2007). Second, all histones of the nucleosome, H2A, H2B, H3, and H4, are highly decorated with a myriad of chemical modifications, most commonly at their N-terminal tails (Bannister and Kouzarides, 2011). These modifications are catalyzed by “writer” enzymes and recognized by “reader” proteins to elicit different responses, including chromatin relaxation or compaction, transcriptional activation or repression, and modulation of co-transcriptional RNA processing. Histone modifications are reversible and can be removed by “eraser” enzymes. Third, nucleosomes are highly dynamic and chromatin remodeling factors can modulate the density and positioning of nucleosomes. Finally, nucleosomes are further assembled into higher-order chromatin structures, including euchromatin, heterochromatin, loops, and topologically associated domains (Pombo and Dillon, 2015). These different levels of epigenetic mechanisms can influence one another. For example, histone modifications can alter the compaction of DNA and/or recruit chromatin-binding proteins (Bannister and Kouzarides, 2011). DNA methylation prevents the addition of some histone modifications associated with active transcription (Okitsu and Hsieh, 2007).

All of these epigenetic mechanisms are known to regulate transcription. For example, DNA methylation at promoters is known to repress transcription, in part by preventing transcription factors from binding to DNA (Razin and Riggs, 1980; Comb and Goodman, 1990). DNA methylation also occurs in gene bodies, including introns, but its functions are less well defined. In addition, specific histone marks correlate with active or inactive transcription (Bannister and Kouzarides, 2011). For example, tri-methylation of histone H3 at lysines 4 and 36, represented as H3K4me3 and H3K36me3, respectively, are associated with actively transcribed genes, while H3K9me2/3 are often found at silenced chromatin regions (Barski et al., 2007). The extent and type of DNA and histone modifications, and the density and positioning of nucleosomes all contribute to controlling DNA accessibility across the genome (Miranda and Jones, 2007; Klemm et al., 2019). Higher DNA accessibility allows transcription factors and other DNA-binding proteins to bind DNA and activate or repress transcription (Klemm et al., 2019). DNA accessibility also alters the rate of transcription by RNA Polymerase II (RNAPII; Jimeno-González et al., 2015). Given that mRNA processing occurs co-transcriptionally, epigenetic mechanisms also play important roles in regulating these events. Indeed, the roles of epigenetic factors in splicing regulation have been extensively studied and a number of excellent reviews are available on this topic (Luco et al., 2011; Brown et al., 2012). Here we will focus on discussing recent advances in understanding the crosstalk between APA and epigenetics.

## APA Regulation by Transcription

As a number of epigenetic factors may regulate APA indirectly *via* modulating transcription, we will begin by discussing the links between transcription and APA. The processes of transcription and mRNA 3'-end processing are tightly coupled. mRNA 3'-end processing factors are recruited to the transcription machinery as early as the pre-initiation complex and are believed to traverse the gene body with RNAPII (Dantonel et al., 1997). Additionally mRNA 3'-end processing is required for transcription termination. PAS recognition by the mRNA 3'-end processing machinery may induce conformational changes in the elongating RNAPII complex that cause termination (Rosonina et al., 2006). Or according to the “torpedo” model, RNA cleavage by the mRNA 3'-end processing machinery generates a 5'-OH end for the nascent RNA, which is degraded by the exoribonuclease Xrn2/Rat1p to induce termination (Rosonina et al., 2006). In both models, mRNA 3'-end processing machinery plays an essential role. How does transcription impact APA? Bioinformatic analyses revealed that highly expressed genes tend to harbor shorter 3'UTRs while lowly expressed genes tend to contain longer 3'UTRs, suggesting that transcription may influence PAS selection (Ji et al., 2011). Although increased RNA stability of isoforms with shorter 3'UTRs could in part explain their increased abundance, as has been demonstrated in several studies (Mayr and Bartel, 2009), Ji and colleagues provided evidence that transcription itself may play a direct role in PAS selection. Using reporter assays, they found that stronger promoters favor the selection of upstream/proximal PAS while weaker promoters favor downstream PAS. In keeping with these results, transcriptional activators have been shown to enhance co-transcriptional mRNA 3'-end processing *in vitro* (Nagaike et al., 2011) and *in vivo* (Rosonina et al., 2003). Stimulation of mRNA 3'-end processing activity by transcription is dependent on the C-terminal domain (CTD) of RNAPII (Rosonina et al., 2003). Mechanistically, transcriptional activation promotes the recruitment of mRNA 3'-end processing factors downstream of the PAS, but not at the promoter region (Glover-Cutter et al., 2008). This suggests that transcriptional activation does not increase recruitment of these mRNA 3'-end processing factors at the start of transcription but rather later, perhaps once the PAS has been transcribed (Glover-Cutter et al., 2008). It is currently unclear how transcriptional activation or promoter sequence could influence downstream events at the 3' end of genes. In addition, enhancers have been recently shown to stimulate cleavage at weak and proximal PAS (Kwon et al., 2021), although the underlying mechanism remains unknown. Nevertheless, these results provided strong evidence that transcriptional activity can profoundly influence mRNA 3'-end processing and APA.

In addition to transcriptional regulation at promoters, RNAPII elongation is also intimately linked to mRNA 3'-end processing. G-rich sequences that cause RNAPII pausing were shown to activate polyadenylation *in vitro* (Yonaha and Proudfoot, 1999). RNAPII is known to pause at PAS and the extent of this pausing may be dynamically regulated to influence APA (Glover-Cutter et al., 2008; Fusby et al., 2015). Increased RNAPII pausing correlates with increased usage of the proximal PAS in the IgM gene (Peterson et al., 2002). The underlying mechanism may again involve the RNAPII CTD. Ser5 phosphorylation in the CTD is enriched at the promoter regions, Ser2 phosphorylation is associated with elongating RNAPII, and Thr4 phosphorylation mainly occurs in the termination zone (Hsin and Manley, 2012). Inhibition/depletion of the kinases and phosphatases responsible for these phosphorylation events, including Cdk12, PP1, and PP2A, have been shown to both disrupt RNAPII elongation and termination, and alter APA (Dubbury et al., 2018; Cortazar et al., 2019; Huang et al., 2020). Given the role of transcription initiation and elongation in APA regulation, any epigenetic factors that alter transcription are predicted to impact APA.

## APA Regulation by DNA Modifications

As mentioned earlier, DNA methylation is a hallmark of silenced chromatin regions and DNA methylation in promoters directly represses transcription. Evidence of direct regulation of APA by DNA methylation came from genomic imprinting studies. Genomic imprinting describes the phenomenon of differential gene expression from the maternal and paternal alleles (Wood et al., 2008; Tucci et al., 2019). Approximately 200 mammalian genes are imprinted and most of them are located in clusters, which share *cis*-regulatory elements to maintain their biased allelic expression (Wood et al., 2008; Tucci et al., 2019). Because imprinted genes are exposed to the same concentration and repertoire of *trans*-acting factors, epigenetic differences, such as DNA methylation, play a critical role in their regulation. Differential DNA methylation was shown to influence allele-specific APA of the imprinted gene *H13* in mice and ultimately establish an imprinted expression pattern (Wood et al., 2008). Within an intron of *H13* and downstream of two *H13* intronic PAS is the promoter for the *Mcts2* gene, which is highly methylated only on the maternal allele. This allele-specific DNA methylation of the *Mcts2* promoter appears to prevent the usage of the intronic *H13* PAS in cis. Utilization of the intronic *H13* PAS on the paternal allele results in expression of a truncated and likely non-functional H13 protein (Wood et al., 2008). A similar mode of regulation was reported for the imprinted retrogene *Nap1l5* in mouse brain, which is expressed from the paternally inherited allele (Monk et al., 2011; Cowley et al., 2012). *Nap1l5* is located within an intron of the *Herc3* gene and downstream of two intronic *Herc3* PAS. In addition, *Nap1l5* is transcribed in the antisense direction of *Herc3*. DNA methylation of a CpG island within the promoter of *Nap1l5* on the maternal allele appears to: (1) prevent usage of the intronic *Herc3* PAS and (2) block expression of *Nap1l5* on the maternal allele (Cowley et al., 2012). This has been attributed to transcriptional interference – an incompletely understood phenomenon in which transcription of one gene represses that of another (Shearwin et al., 2005; Cowley et al., 2012).

**Figure 2:**
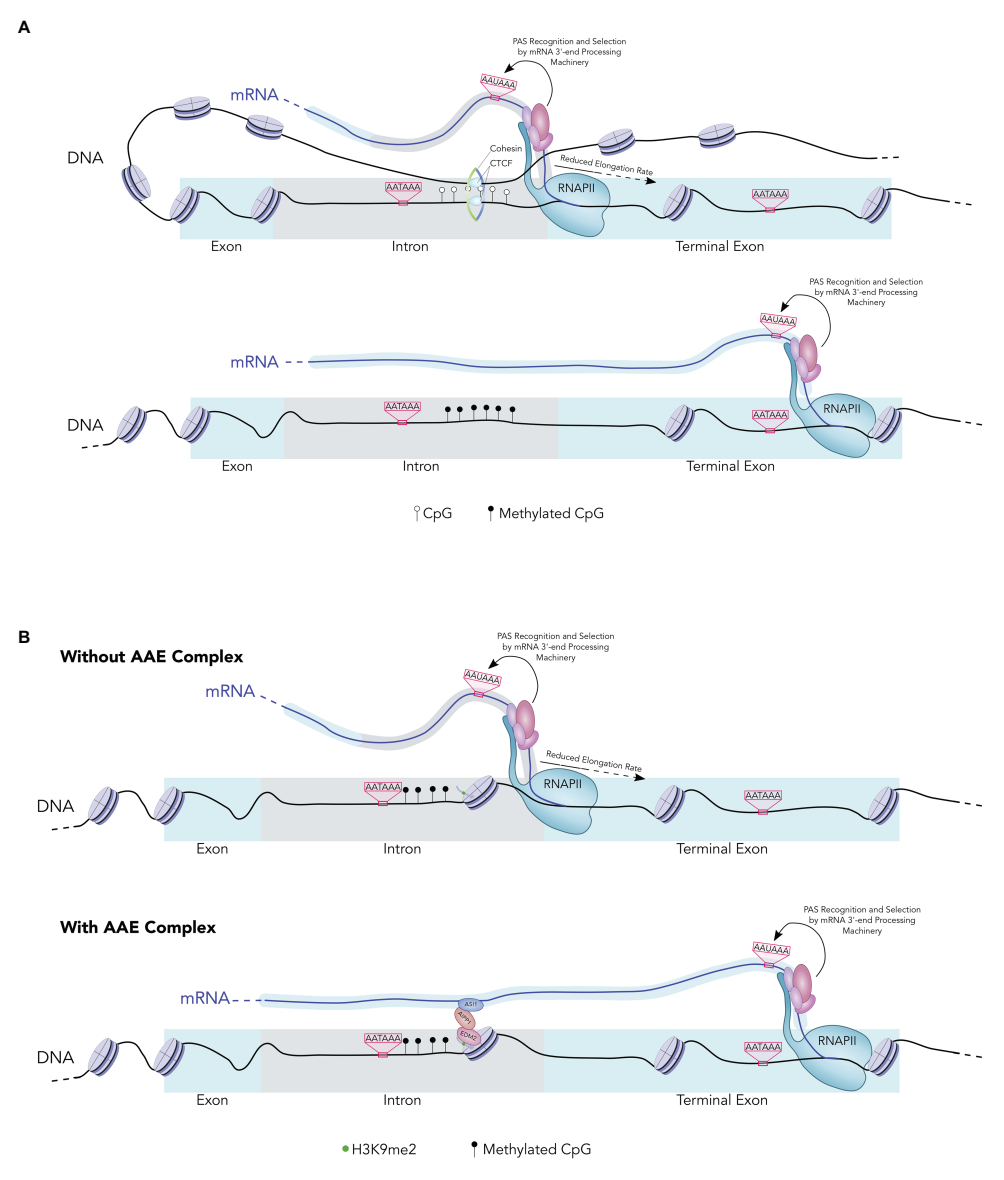
**(A)** A model of APA regulation by DNA methylation. DNA is wrapped around nucleosomes (purple). In the top example, an unmethylated intronic CpG island allows the Cohesin-CTCF complex to bind downstream of an intronic PAS. The PAS are represented by AATAAA. Cohesin-CTCF binding to DNA forms a DNA loop and enhances RNA Polymerase II (RNAPII) pausing. This increases usage of the intronic PAS. In the bottom example, the intronic CpG island is highly methylated and the Cohesin-CTCF complex cannot bind. As a result, RNAPII does not pause downstream of the intronic PAS, the intron is removed by splicing, and the downstream PAS is selected in the terminal exon. **(B)** A model of APA regulation by the AAE complex. In the top example, intronic heterochromatin leads to RNAPII pausing in the absence of the AAE complex. This increases usage of the intronic PAS. In the bottom example, the AAE complex binds the heterochromatic region and counteracts the effects of heterochromatin on RNAPII pausing. RNAPII then transcribes the downstream PAS and this PAS is recognized and selected by the mRNA 3'-end processing machinery.

DNA methylation is known to regulate alternative splicing in a similar manner and CTCF plays a key role in this process (Shukla et al., 2011). CTCF specifically binds to unmethylated DNA and DNA-bound CTCF causes RNAPII pausing, thereby activating nearby splice sites. The same mechanism also underlies DNA methylation-mediated APA regulation. CTCF binds to unmethylated CpG islands within introns to recruit the cohesin complex and enhance RNAPII pausing, which in turn promotes the usage of nearby intronic PAS (Figure 2A; Nanavaty et al., 2020). This mechanism is likely to be responsible for generating the differential APA patterns of imprinted genes.

## APA Regulation by Histone Modifications and Chromatin Structure

Assembly of DNA into nucleosomes and chromatin alters DNA accessibility and creates physical barriers for the transcription machinery. Indeed, an *in vitro* study using the bacteriophage T7 RNA polymerase, under conditions in which its transcriptional rate was similar to eukaryotic RNAPII, found that the presence of nucleosomes decreases the elongation rate by increasing pausing at DNA-encoded pause sites (Protacio et al., 2000). Nucleosome occupancy levels and positioning are not random. PAS-encoding DNA regions are generally depleted of nucleosomes and there is a positive correlation between nucleosome depletion surrounding the PAS and their usage (Spies et al., 2009). Interestingly, despite being depleted of nucleosomes, PAS regions generally display low DNA accessibility as measured by DNase I sensitivity (Ji et al., 2011; Lee and Chen, 2013). The overall low DNA accessibility near PAS and enrichment of nucleosomes downstream may stimulate RNAPII pausing to allow for PAS recognition and mRNA 3'-end processing at these sites.

PAS are also associated with specific histone marks. Higher levels of H3K4me3 and H3K36me3, both marks of actively transcribed genes, are observed near highly used PAS (Barski et al., 2007; Spies et al., 2009; Ji et al., 2011). These observations indicate that nucleosome and histone modifications are linked to mRNA 3'-end processing. In support of this, the Moore laboratory recently showed that genetic ablation of *SET1* and *SET2*, which encode the enzymes responsible for H3K4me3 and H3K36me3, respectively, alters APA of many tested genes (Kaczmarek Michaels et al., 2020). At the molecular level, *SET1* and *SET2* deletion decreases nucleosome occupancy levels near PAS and Ser2 phosphorylation of the RNAPII CTD (Kaczmarek Michaels et al., 2020). Further, a recent report in *Arabidopsis* showed genetic inactivation of *hda6*, a gene encoding an enzyme that deacetylates histones, activated usage of certain PAS (Lin et al., 2020). PAS with increased usage in the *hda6* mutant were located closer to H3K9ac and H3K14ac peaks than in wild-type cells (Lin et al., 2020). This suggests loss of HDA6 increases acetylation at these sites and promotes the usage of the nearby PAS. Although the cause-effect relationship among all of these molecular changes remains unclear, these studies provided genetic evidence that histone modifications play an important role in APA regulation.

In addition to the genome-wide associations, recent studies have also provided gene/sequence-specific examples between histone modifications and APA regulation. For example, transposable elements and repeat elements (TREs) are highly prevalent in eukaryotic genomes. They are typically found in clusters and the chromatin regions containing TREs are generally silenced through DNA methylation and repressive histone modifications such as H3K9 methylation (Slotkin and Martienssen, 2007). Interestingly, many TREs bearing these repressive chromatin signatures are found in the introns of protein-coding genes (van de Lagemaat et al., 2003). These intragenic heterochromatin regions contribute to RNAPII pausing and promotes usage of proximal PAS (Neve et al., 2016). Interestingly, several recent studies have identified a multi-protein complex, called the AAE complex, which counteracts the effect of heterochromatin on transcription and APA (Duan et al., 2017). The AAE complex consists of at least three subunits: ASI1, AIPP1, and EDM2. ASI1 is a plant-specific protein that contains an RNA-recognition motif (RRM) and a bromo-adjacent homology (BAH) domain (Duan et al., 2017). EDM2 is a chromatin regulator that harbors three plant homeodomains (PHDs), which are known to bind to H3K9me2 and other histone marks (Lei et al., 2014; Duan et al., 2017). Finally AIPP1 is an RRM-containing protein that bridges ASI1 and EDM2 (Duan et al., 2017). According to the current model, the AAE complex binds to intronic TRE-containing heterochromatin at least in part *via* EDM2 and prevents the stalling of RNAPII (Figure 2B). In the absence of this complex, increased RNAPII pausing near the intragenic heterochromatin leads to activation of intronic PAS and thus the production of truncated mRNAs of the host genes (Figure 2B). Mechanistically this may be similar to the DNA methylation-mediated APA regulation in that both histone and DNA modifications modulate PAS selection indirectly by controlling RNAPII elongation rate.

## Regulation of Chromatin Structure by mRNA 3’-End Processing

Most studies related to epigenetics and APA have focused on regulation of APA by epigenetic mechanisms, but recent evidence highlights modulation of epigenetics by APA. In 2006, the Yamanaka group demonstrated that differentiated cells can be reprogrammed to a stem cell-like state by over-expressing four genes (Takahashi and Yamanaka, 2006). The efficiency of this process, however, is very low, and it was postulated that there are genes that block somatic reprogramming. Interestingly, the mRNA 3'-end processing factor CFIm25/Nudt21 was recently identified as such a roadblock gene (Brumbaugh et al., 2017). CFIm25 is a subunit of the CFIm complex, which is a sequence-dependent activator of mRNA 3'-end processing (Zhu et al., 2017). It binds to an enhancer sequence, UGUA, and promotes the recruitment of the core mRNA 3'-end processing machinery. Due to the enrichment of the UGUA enhancer sequence at distal PAS of many genes, CFIm promotes the usage of these PAS and the production of mRNAs with longer 3' UTRs (Zhu et al., 2017). Importantly, knockdown of CFIm25 in somatic cells leads to 3' UTR shortening of over 1,000 genes, including a number of chromatin regulators (Brumbaugh et al., 2017). Such APA changes lead to the upregulation of these chromatin regulators, which in turn result in more efficient removal of the differentiation-associated chromatin landscape and faster re-establishment of stem cell-specific chromatin signatures. Given that CFIm25 was also shown to suppress glioblastoma (Masamha et al., 2014), APA-mediated regulation of chromatin structure may play a role in tumorigenesis.

Changes in mRNA 3'-end processing can also physically disrupt 3D genome organization. Influenza virus infection leads to host gene shut-off. One mechanism by which the virus inhibits host gene expression is by inhibiting host mRNA 3'-end processing *via* the viral protein NS1 (Nemeroff et al., 1998). A recent study demonstrated that such inhibition of mRNA 3'-end processing also leads to genome-wide transcription termination defects (Zhao et al., 2018). Elongating RNAPII may move past the normal termination sites by hundreds of kilobases and displace DNA-bound CTCF along the way, thereby disrupting chromatin looping (Heinz et al., 2018). Similarly, herpes simplex virus 1 also inhibits host mRNA 3'-end processing and transcription termination, resulting in a breakdown of the 3D genome organization of the host cells (Rutkowski et al., 2015; Hennig et al., 2018; Wang et al., 2020). These studies clearly demonstrate that mRNA 3'-end processing and APA can regulate the global chromatin structure through multiple mechanisms.

## Discussion

APA continues to gain appreciation as a major strategy used by cells to fine-tune gene expression. Researchers are increasingly mapping APA patterns and studying its regulatory mechanisms across organisms, cell types, and during cell fate transitions. Recent advances in the field have clearly demonstrated that epigenetic mechanisms, including DNA and histone modifications and chromatin structures, play an important role in APA regulation. Mechanistically, many of these epigenetic factors regulate APA indirectly through modulating RNAPII elongation and pausing. For future studies, it will be critical to identify and characterize the factors that mediate the communication between DNA/chromatin and RNA processing. For splicing, a number of splicing regulators have been shown to bind to specific histone mark readers, thereby mediating the regulation of splicing by chromatin features. Such interactions are currently lacking for APA regulation. For example, in the AAE complex mentioned earlier, EDM2 recognizes histone marks and ASI1 most likely binds to RNA, thereby linking chromatin directly to RNA. Future studies will determine if similar complexes also exist in metazoans. Finally additional efforts are needed to understand the biological consequences of epigenetics-mediated APA regulation in development and in diseases.

## Author Contributions

LS and YS contributed to the writing of this article. Both authors contributed to the article and approved the submitted version.

### Conflict of Interest

The authors declare that the research was conducted in the absence of any commercial or financial relationships that could be construed as a potential conflict of interest.
